# Minimally invasive treatment of uterine necrosis with favorable outcomes: an uncommon case presentation and literature review

**DOI:** 10.1186/s12905-024-03089-w

**Published:** 2024-04-27

**Authors:** Tengge Yu

**Affiliations:** Department of Gynecology and Obstetrics, West China Xiamen Hospital of Sichuan University, Xiamen, 361000 China

**Keywords:** Necrosis, Minimally invasive surgical procedures, Fertility preservation, Bacterial infections

## Abstract

**Background:**

Uterine necrosis is a rare condition and is considered a life-threatening complication. However, cases of uterine necrosis were rarely reported, particularly those caused by infection. In terms of treatment, no minimally invasive treatment for uterine necrosis has been reported, and total hysterectomy is mostly considered as the treatment option.

**Objective:**

The article specifically focuses on minimally invasive treatments and provides a summary of recent cases of uterine necrosis.

**Case presentation:**

We report the case of a 28-year-old patient gravid 1, para 0 underwent a cesarean section after unsuccessful induction due to fetal death. She presented with recurrent fever and vaginal discharge. The blood inflammation markers were elevated, and a CT scan revealed irregular lumps with low signal intensity in the uterine cavity. The gynecological examination revealed the presence of gray and white soft tissue, approximately 5 cm in length, exuding from the cervix. The secretions were found to contain Fusobacterium necrophorum, Escherichia coli, and Proteus upon culturing. Given the patient’s sepsis and uterine necrosis caused by infection, laparoscopic exploration uncovered white pus and necrotic tissue openings in the anterior wall of the uterus. The necrotic tissue was removed during the operation, and the uterus was repaired. Postoperative pathological findings revealed complete degeneration and necrosis of fusiform cell-like tissue. Severe uterine necrosis caused by a multi-drug resistant bacterial infection was considered after the operation. She was treated with antibiotics for three weeks and was discharged after the infection was brought under control. The patient expressed satisfaction with the treatment plan, which preserved her uterus, maintained reproductive function, and minimized the extent of surgery.

**Conclusion:**

Based on the literature review of uterine necrosis, we found that it presents a potential risk of death, emphasizing the importance of managing the progression of the condition. Most treatment options involve a total hysterectomy. A partial hysterectomy reduces the extent of the operation, preserves fertility function, and can also yield positive outcomes in the treatment of uterine necrosis, serving as a complement to the overall treatment of this condition.

**Supplementary Information:**

The online version contains supplementary material available at 10.1186/s12905-024-03089-w.

## Background

Uterine necrosis is a rare complication. Several cases of uterine necrosis have been reported following embolization of the uterine arteries for postpartum hemorrhage or uterine fibroids, or as a result of severe endometritis [1]. Symptoms of uterine necrosis caused by infection typically include lower abdominal pain, fever, and foul-smelling vaginal discharge. When the infection affects the tissue surrounding the uterus, the uterus becomes enlarged and tender, and the edema of the inflamed tissue holds the uterus in place. Some complications may occur infrequently, including peritonitis, pelvic vein thrombosis, pulmonary embolism, pelvic abscess, sepsis, kidney damage, and even death. Diagnosis is usually based on clinical symptoms and physical examination. Inflammatory markers, imaging studies, and secretion cultures can also assist in the diagnosis. Hysteronecrosis is typically treated with a total hysterectomy. Most patients recover, and only a small number of patients do not survive. We reported a case of uterine necrosis caused by infection. We removed part of the uterus instead of performing a total hysterectomy. The patient recovered well. Few cases of uterine necrosis have been reported, and no one has reported minimally invasive treatment for it. Given the rarity of the case and the lack of minimally invasive treatment options for uterine necrosis, this report was written in conjunction with a literature review summarizing similar cases of uterine necrosis.

## Case presentation

We report the case of a 28-year-old patient who was gravid 1, para 0, with no significant medical history. The patient is Asian, from the Han ethnic group, China’s largest ethnic group. When she was 32 weeks pregnant, intrauterine fetal demise was confirmed by ultrasound. A cesarean section was performed due to the difficulty of vaginal trial labor following a lateral perineal incision, which was necessary because the fetus’s shoulder was exposed. After the operation, the patient continued to experience a high fever, with a maximum temperature of 39.5 degrees Celsius, and the fat around the abdominal incision has become liquefied. The number of patient’s pulses was 140, respiratory rate was 22 times per minute, and blood pressure was 131/87mmhg. After receiving treatment with medications such as Tienam and Piperacillin, the patient’s body temperature and inflammation returned to normal, and she was discharged from Municipal integrated traditional Chinese and Western medicine hospital. The type of antibiotic Tienam is Carbapenem antibiotics, and the dose is 500 mg by injection three times a day. The type of antibiotic Piperacillin is semi-synthetic penicillin antibiotics, and the dose is 1.5 g by injection three times a day. The disease subside after 7 days treatment.

A week later, she was admitted to Municipal integrated traditional Chinese and Western medicine hospital for the second time due to fever and pain in her lower left abdomen. The patient’s heart rate was normal, respiratory rate was 20 times per minute, and blood pressure was 121/80mmhg. The blood inflammation index was elevated, indicated by a C-reactive protein level of 52.01 mg/L. Brain CT and lung CT scans revealed no significant abnormalities. She was discharged after two weeks of treatment with medications such as Tienam and Piperacillin with the same dose as last time. The disease subside after 5 days treatment.

**Fig. 1:**
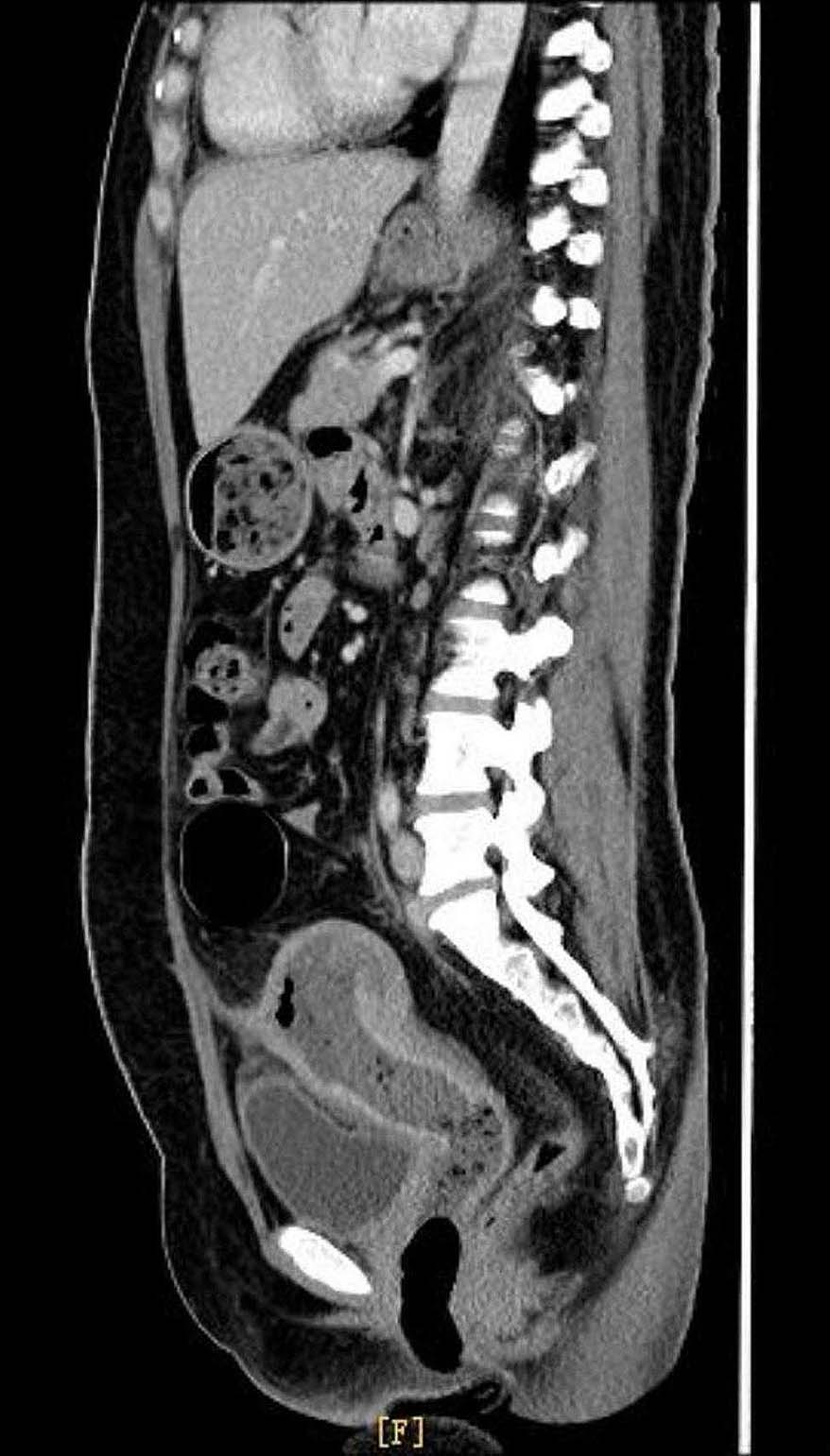
Sagittal computed tomography images. The uterus shows heterogeneous spongiform enlargement with multiple air locules, measuring 4 × 2.6 cm and extending over 5 cm. We have observed a difficulty in distinguishing between the myometrium and the endometrial cavity

Five days later, she was admitted to our hospital for the third time due to a recurring fever, accompanied by vaginal purulent discharge and odor. The patient’s heart rate was 110 times per minute, respiratory rate was 23 times per minute, and blood pressure was 132/85mmhg. There was no increase in β-HCG, white cell count was 12.3 × 10^9 /L in the differential blood count, hemoglobin was 104 g/L, and procalcitonin was 0.12 ng/ml. She felt feverish and lethargic, with mild nausea. The patient was treated orally with Moxifloxacin by 1 tablet once a day for 3 days. A vaginal color ultrasound revealed a hypoechoic area in front of the uterus, indicating encapsulated effusion. The ultrasound also revealed an abnormal uterine echo, uneven uterine enlargement with abundant blood supply, trace effusion of the cervical canal, and pelvic effusion. The enhanced CT scan revealed swelling and adhesion of the anterior wall of the uterus and the adjacent anterior abdominal wall, along with changes in the surrounding exudate. Additionally, a lumpy, uneven low signal shadow was observed in the uterine cavity, along with visible pelvic fluid (Fig. 1). The histopathological analysis of intrauterine effluents revealed degenerative smooth muscle tissue accompanied by pus. Anaerobic culture of cervical secretions suggested the presence of Fusobacterium necrophorum. Biopsy of cervical and vaginal lesions revealed complete necrosis of fusiform cell-like tissue, with increased infiltration of inflammatory cells, and no identifiable endometrial tissue. Due to the presence of pus in the uterus, morinidazole was administered, and uterine drainage was performed. However, the result was not favorable. A gynecological examination revealed the presence of necrotic tissue in the vagina, extending approximately 5 cm from the cervical opening. The tissue appeared white and emitted a foul odor. It was recommended to undergo a laparoscopic surgery.

**Fig. 2:**
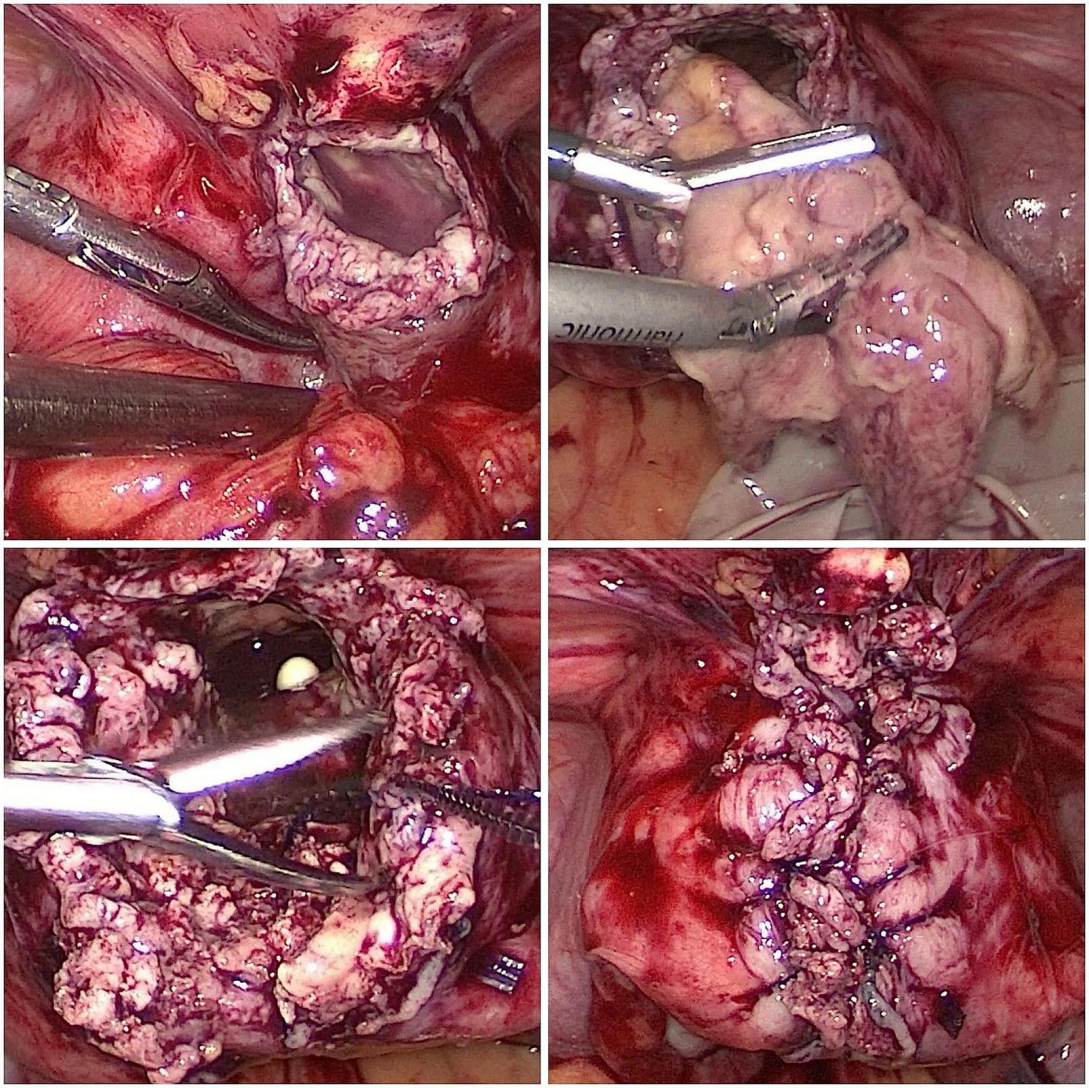
The images seen during the surgery. The necrotic tissue of the uterus, attached to the anterior wall of the abdomen, is clearly visible in gray and white colors. It is situated in the anterior wall of the uterus and is connected to the uterine cavity

**Fig. 3:**
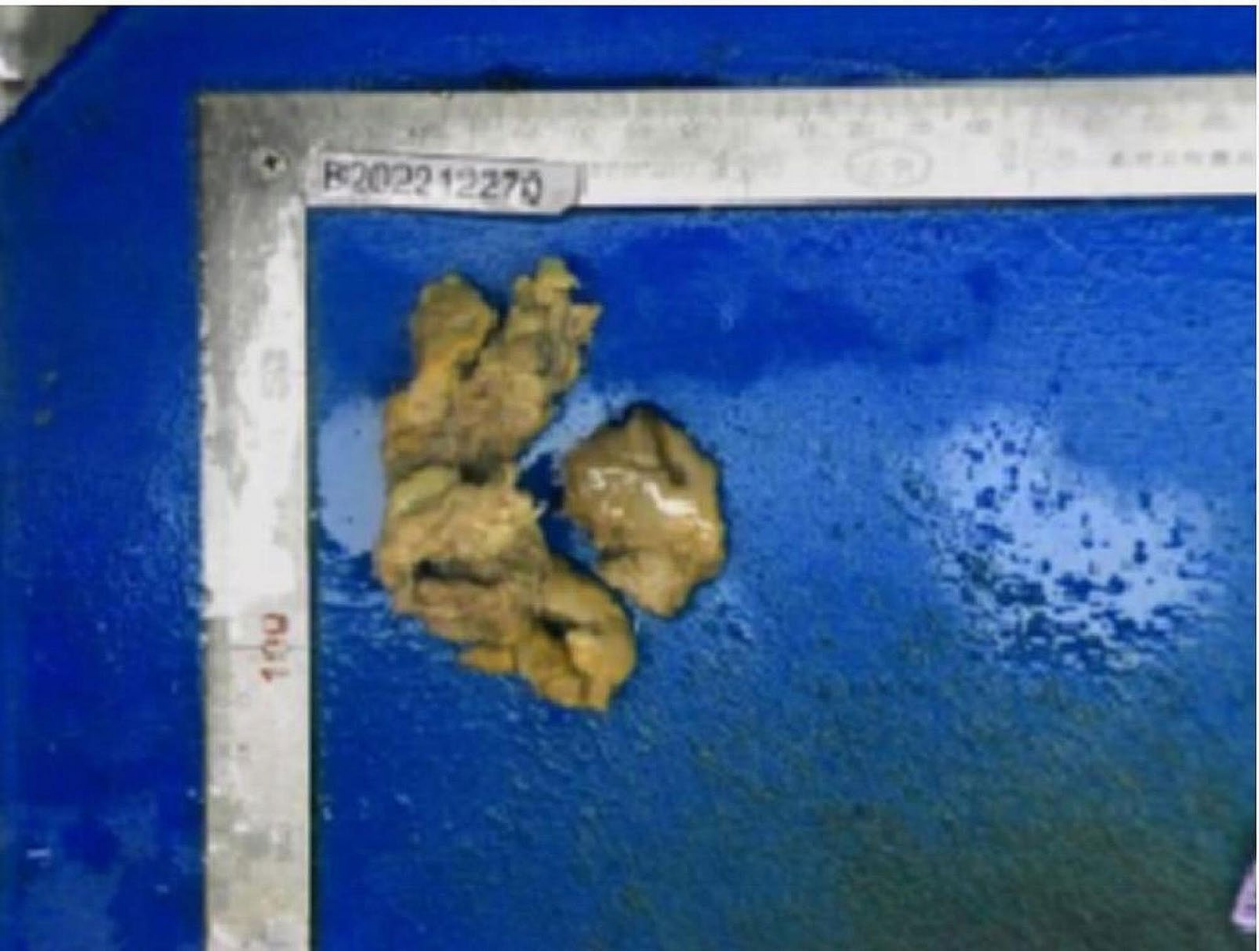
The gross specimen appeared gray in color, with an extremely soft texture, measuring about 5 cm in length, and accompanied by a foul odor

Given that the patient had strong fertility requirements, the surgical procedure included laparoscopic necrotic tissue excision, uterine reconstruction, and the uterine drainage tube placement. During the laparoscopy, adhesion of the uterus to the anterior wall of the abdomen was observed. An opening with white pus and necrotic tissue was visible in the anterior wall of the uterus. The necrotic tissue in the cavity was removed during the operation (Fig. 2 Fig. 3). Cefoxitin (1.5 g tid ivgtt) and ornidazole (500 mg bid oral) were administered postoperatively to prevent infection for 2 days. After the surgery, the patient developed a fever with a peak body temperature of 39.3 degrees Celsius, which prompted a switch to cefoperazone-sulbactam sodium (2 g bid ivgtt) and ornidazole antibiotics (500 mg bid oral) for 7 days. After the body temperature returned to normal, the antibiotics were downgraded, the uterine drainage tube was removed, and oral antibiotics were continued after discharge. The results of the vaginal secretion culture indicated the presence of Escherichia coli and Proteus bacteria. The pathological results revealed extensively denatured necrotic tissue with calcification and heightened inflammatory cell infiltration.

## Discussion

Uterine necrosis is a rare and serious complication. Cases of uterine necrosis have been reported in the literature as a complication of cesarean section, embolization for postpartum hemorrhage, or for a leiomyomatous uterus, as well as in cases of severe endometritis [2–7] (Table 1). Several authors have described cases of uterine necrosis associated with intrapartum or postpartum complications that increase the risk of infection. The literature reports cases of uterine necrosis resulting from the placement of B-Lynch compression sutures, uterine artery embolization, or surgical ligation techniques used to treat postpartum hemorrhage. These procedures may increase the risk of infection as the tissue becomes devascularized. A. Fouad et al. described a case similar to ours in which a patient underwent a cesarean section due to fetal death, followed by postoperative sepsis and purulent uterine necrosis. Despite undergoing a hysterectomy, the disease continued to progress and ultimately resulted in death due to septic shock and multiple organ failure.

**Table 1:** Cases of uterine necrosis

Author and date	Age, G.P., delivery type	Cause of Uterine necrosis	Treatment	Outcome
A. Fouad et al. (2023)	30, G4P3, Cesarean delivery	Bacterial infection	Total hysterectomy with bilateral salpingo-oophorectomy and broad-spectrum antibiotic therapy	Died due to septic shock with multiple organ failure
Emily Lin et al. (2022)	49, G1P0010	Laparoscopic Radiofrequency Myoma Ablation	Total hysterectomy and broad-spectrum intravenous antibiotics postoperatively	Recover and discharge
Linfeng Luo et al. (2021)	28, unknow, Cesarean delivery	B-Lynch suturing for postpartum hemorrhage	Subtotal hysterectomy and resection of necrotic uterus adnexa	Temperature returned to normal
Mariam Riad et al. (2020)	41, G7P6, Vaginal delivery	Group A streptococcal infection	Penicillin, clindamycin, and IV immunoglobulin therapy	Multi-organ infarctions, acute respiratory distress syndrome, and severe reactive arthritis
Joan Tymon-Rosario et al. (2019)	29, G1P0, Cesarean delivery	Escherichia coli infection	Total abdominal hysterectomy, bilateral salpingectomy, and Jackson Pratt drain placement	Healed completely
Kei Tanaka et al. (2017)	40, unknow, Cesarean delivery	Uterine artery embolization for post-partum hemorrhage	Total hysterectomy	Recover and discharge
David Kashan et al. (2016)	72, G2P2, Unknow	Intra-Abdominal Clostridium perfringens Gas Gangrene	Broad-spectrum antibiotic, total hysterectomy, bilateral salpingectomy, and enterectomy	Died of multiple organ failure resulting from sepsis
T Widelock et al. (2015)	19, G4P0212, Cesarean delivery	Fusobacterium necrophorum Infection	Vancomycin, meropenem, and total hysterectomy	Complicated with pulmonary abscess, pelvic abscess, and renal failure

The case we report has identified pathogenic bacteria in the culture, which is significant for diagnosing infection-induced uterine necrosis. Fusobacterium necrophorum is a pleomorphic, Gram-negative, non-spore-forming obligate anaerobic coccobacillus. It is associated with localized abscesses, throat infections, and life-threatening systemic diseases. It is a common resident of the oral cavity and the vagina. Of the two subspecies of Fusobacterium necrophorum, biovar B is the primary pathogen for humans. Potential virulence factors include cell wall endotoxin lipopolysaccharide, hemagglutinin, and hemolysin. Most reported cases related to gynaecology occurred in the postpartum or post-abortion period, in addition to a few reports associated with the use of intrauterine devices, tubo-ovarian abscesses, and gynecological Lemierre’s syndrome [8–10]. Although infected with the same pathogen, the case reported by T. Widelock et al. developed more severe symptoms, including lung abscesses and kidney failure, as a result of hematoplasm infection [11–13].

A pelvic ultrasound is the initial diagnostic test that can reveal signs of uterine necrosis. The uterine cavity is typically expanded and exhibits multiple echogenic foci with accompanying dirty acoustic shadowing. Little or no vascularity is observed [14]. The diagnosis requires further exploration through a CT scan or MRI, as these are the preferred methods of investigation. The CT scan is highly useful for diagnosis as it demonstrates the presence of gas in the myometrium, the lack of enhancement of the myometrium after contrast injection associated with uterine enlargement, and the presence of free fluid in the peritoneal space [15, 16].

Since uterine necrosis is described as a life-threatening complication, it is suggested to manage it with hysterectomy and broad-spectrum antibiotic therapy [17, 18]. But sometimes it’s a case-by-case situation.

- Avoid the chances of associated infections by systematic vaginal sampling in the third trimester, and promote good asepsis during surgery and antibiotic coverage in case of doubt about any undiagnosed prepartum infection, which may potentiate hypoxia and the risk of necrosis. Uterine necrosis may be secondary to all these intertwined factors and could be potentiated by an environment of hypoxia, hypoperfusion, hypovolemia secondary to hemorrhage, massive transfusions with disadvantages in a patient who is immunocompromised by pregnancy, and possibly, by other vitamin and iron deficiencies.
- Given the limited number of reported cases of uterine necrosis in the past, there is no standardized treatment protocol. However, due to the potential fatality of uterine necrosis, most treatment options involve total hysterectomy. In our case, only the necrotic tissue of the uterus was removed in young women who had not given birth, and the prognosis for the patient is good. This study also has limitations, including the short follow-up time and the small number of cases collected. It needs to be complemented by subsequent case reports related to uterine necrosis.

## Conclusion

Based on the literature review of uterine necrosis, we found that it presents a potential risk of death, emphasizing the importance of managing the progression of the condition. Most treatment options involve a total hysterectomy. A partial hysterectomy reduces the extent of the operation, preserves fertility function, and can also yield positive outcomes in the treatment of uterine necrosis, serving as a complement to the overall treatment of this condition.

### Electronic supplementary material

Below is the link to the electronic supplementary material.

Supplementary Material 1

## Data Availability

No datasets were generated or analysed during the current study.
